# Sexually Transmitted Infections and Reproductive Health Morbidity in a Cohort of Female Sex Workers Screened for a Microbicide Feasibility Study in Nellore, India

**DOI:** 10.5539/gjhs.v5n3p139

**Published:** 2013-02-24

**Authors:** Avina Sarna, Barbara A. Friedland, Aylur K. Srikrishnan, Lauren L. Katzen, Waimar Tun, Sharon A. Abbott, Ulrike Rawiel, Christine A. Kelly, C. S. Shalini, Suniti Solomon, Barbara S. Mensch

**Affiliations:** 1 Population Council, Delhi, India; 2 Population Council, New York, USA; 3 YRG Care, Chennai, India; 4 Population Council, Washington D.C., USA; 5 YRG Care, Nellore, India

**Keywords:** female sex workers, microbicides, HIV prevention, sexually transmitted infections, reproductive health

## Abstract

**Short Summary:** Four-month placebo vaginal gel trial conducted to determine the feasibility of recruiting FSWs for future Phase 2/3 microbicide trials. RH morbidity and HIV and STI prevalence are reported.

Women constitute 38% of India’s 2.4 million HIV-infected persons. Microbicides are potential HIV-prevention products currently undergoing clinical trials for efficacy. A four-month placebo vaginal gel trial was conducted in Nellore, India to determine the feasibility of recruiting a suitable cohort of female sex workers (FSWs) for a future vaginal microbicide efficacy trial. We report on the HIV and STI prevalence and reproductive health (RH) morbidity of FSWs screened for the trial. Results: 529 FSWs completed screening procedures; of those 33.6% were found ineligible. The mean age was 30.9 years; 68.6% women were married and 57.5% were home-based FSWs. Self-reported symptoms included abnormal vaginal discharge (31.6%), genital itching (3.4%), uterine mass/prolapse (3%) and painful intercourse (2.6%). Gynecological surgery was reported by 73.2% of participants; of those 10.5% had undergone a hysterectomy. Female sterilization was the most commonly reported contraceptive method. Pelvic examination showed vaginal discharge (50.7%), cervical discharge (5.3%), uterine/vaginal wall prolapse (2.6%), and cervical mass/nodule/vesicles/genital warts (4.2%). Common epithelial findings included erythema (79.1%) and vesicles/bullae (6%); 46% of participants had Papanicolaou tests graded as inflammatory and 1.1% as malignant. HSV-2 was the mostly commonly detected STI (60.7%) followed by trichomoniasis (15.5%), HIV (5.3%), syphilis (2.8%), chlamydia (2.2%) and gonorrhoea (0.7%). RTIs were more common: bacterial vaginosis (27.8%) and candidiasis (18.9%). Conclusions: The low HIV prevalence and high RH morbidity in the population makes this site unsuitable for a future phase 2 or 3 microbicide efficacy trial. HIV prevention programs targeting this population should include access to RH services.

## 1. Background

India has an estimated 2.4 million adults living with HIV with a national prevalence of 0.31%; women constitute 38% of this population (UNAIDS, 2010). Recent data indicate that 90% of HIV-positive women in India were infected within long-term relationships, highlighting the need for HIV prevention methods that women could use to protect themselves (UNAIDS, 2010). HIV prevention efforts in India have focused largely on improving awareness, promoting HIV testing, and condom provision. Topical microbicides, products applied to the vaginal or rectal mucosa, are being developed to reduce the sexual transmission of HIV and other infections (Minces & McGowan, 2010); this has the potential to be an ideal prevention product for women in India.

The HIV epidemic in India is largely concentrated in high-risk groups including female sex workers (FSWs) (National AIDS Control Organisation (NACO), 2011). HIV prevalence is significantly higher among FSWs than in the general female population (4.9% vs. 0.3%), despite recent data indicating declining prevalence in FSWs. Therefore, FSWs would be a logical population for a vaginal microbicide effectiveness trial in India (NACO, 2011; Joshi, Solomon, Mayer, & Mehendale, 2005). The eligibility criteria for a clinical trial of an active microbicide product are stringent, and require participants to be in good reproductive health (RH) at enrollment. Accordingly, obtaining suitable cohorts for microbicide trials is often challenging.

Microbicide trials in India have been limited, for the most part, to an assessment of safety and acceptability. Baseline characteristics specifically for an Indian cohort were reported only for the Phase 1 trial of Buffergel conducted in Pune, Maharashtra (van de Wijgert et al., 2001). Similarly, most microbicides studies conducted in India have reported adverse events that occurred during the trial, rather than overall RH at baseline (Damme et al., 2008; Hillier et al., 2008; Joshi et al., 2003; 2005; 2006; van de Wijgert et al., 2001).

Recent studies among FSWs in India have documented HIV prevalence (Brahme et al., 2006; Dandona et al., 2005; *National Summary Report- India, Integrated Behavioural and Biological Assessment (IBBA), Round 2 (2009-2010)*, 2011) that varies from 2.2% (Chennai, Tamil Nadu) to 23.3% (East Godavari, Andhra Pradesh (AP) (IBBA, 2011). Similarly, syphilis prevalence varied from 14.6% (Pune, Maharashtra) to 17.9% in (East Godavari, AP), and prevalence of herpes simplex virus type-2 (HSV-2) ranges between 78.0-88.9% in the same districts (IBBA, 2011). Several studies have evaluated risk factors associated with HIV and sexually transmitted infection (STI) (Dandona et al., 2005; Ramesh et al., 2008; Reed, Gupta, Biradavolu, Devireddy, & Blankenship, 2011; Shannon & Csete, 2010), but few have addressed the RH morbidity and needs of FSWs (Wayal et al., 2011).

We undertook a four-month placebo vaginal gel trial in Nellore, AP to determine the feasibility of recruiting a suitable cohort of FSWs for a future Phase 2/3 microbicide trial. In this paper, we report on HIV and STI prevalence, risk factors associated with these infections, and RH morbidity in this cohort.

## 2. Methods

### 2.1 Study Population

Between January and December 2010, FSWs living or working in and around Nellore were recruited using respondent driven sampling (Tun et al., 2011) and those who completed a community-based survey about willingness to participate (WTP) in a microbicide trial (Mensch et al., 2012) were referred for screening to a clinic operated by the Y.R. Gaitonde Centre for AIDS Research and Education (YRG CARE), a non-governmental organization. FSWs were eligible if they were 18-45 years old; had sex in exchange for money, gifts or other favors at least once in the month before screening; were HIV negative; were willing and able to give informed consent and provide locator information; and did not plan to move out of Nellore district for six months. Women were excluded if they used recreational injectable drugs; were participating in another HIV-prevention study; were pregnant or planning to become pregnant in the next six months; had abnormal genital findings with deep epithelial disruption that did not improve with treatment; had symptomatic genital herpes; had a positive syphilis test (titer >1:32); or had a Papanicolaou (Pap) smear graded as malignant or indicating severe inflammatory changes that did not improve after treatment. FSWs diagnosed with a reproductive tract infection (RTI) were simultaneously treated and enrolled, and those with an STI or inflammatory Pap smear were treated and re-screened.

### 2.2 Clinical and Laboratory Procedures

At screening and monthly visits, women had a urine pregnancy test (ACON hCG™ ACON Laboratories, San Diego, CA, USA). Women underwent a pelvic examination by a doctor at screening and Month 4 where an endocervical sample was collected to test for Chlamydia trachomatis (CT) and Neisseria gonorrhea (NG) using Multiplex PCR DNA (Roche Molecular Systems Inc., Branchburg, NJ, USA), and a vaginal specimen was collected for Trichomonas vaginalis (TV) testing (InPouch™ TV culture unit; Biomed Diagnostics, San Jose, CA, USA). At screening, a Pap smear was done (Touchless One-slide Pap Smear Kit™, Chennai, India) with results interpreted using the Bethesda classification (Solomon et al., 2002). If women were symptomatic at screening or any other visit (based on self-report or clinical findings) a vaginal swab was collected for a wet mount to test for RTIs: Bacterial Vaginosis (BV) and Candida albicans. BV was diagnosed based on Amsel criteria (Amsel et al., 1983). Genital abnormalities were classified by type, location, size, color, shape and degree of epithelial disruption in accordance with the Photo Atlas for Microbicide Evaluation (Bollen, Kilmarx, & Wiwatwongwana, 2002).

Blood samples at screening and Month 4 were tested for HIV, HSV-2 and syphilis. An HIV-negative result was based on one single highly sensitive rapid test, and a positive result was based on two confirmatory tests per National AIDS Control Organization (NACO) guidelines (2006). Rapid HIV tests used in this study were: Determine™ HIV-1/2 (Inverness Medical Japan Co Ltd., Japan), First Response™ HIV Card Test 1-2.O (Premier Medical Corporation Ltd. Daman, India) and Signal™ HIV-1/2 rapid visual spot test (Span diagnostics, Surat, India). A fourth Tridot test (Biomed Industries, Himachal Pradesh, India) was used to confirm HIV-2 infection, if detected on the first rapid test. An anti-HSV-2 ELISA test (EUROIMMUN, Deutschland, Germany) was used to detect HSV-2 antibodies, and Rapid Plasma Reagin (RPR, Span Diagnostics Ltd., Surat, India) and Treponema Pallidum Particle Agglutinin (TPPA) (SERODIA-TPPA, Fujirebio Inc. Tokyo, Japan) were used to confirm active syphilis infection. Rapid HIV tests, pregnancy tests, and wet mounts were done on-site. All other samples were transported to YRG CARE’s main laboratory or to a cytology laboratory (Pap smears) in Chennai. Bacterial STIs and RTIs diagnosed on laboratory tests at screening and during the trial were treated on-site per NACO guidelines (2007); any infection detected in these women at endline was considered a new infection. A new HIV or HSV-2 infection was defined as a positive antibody test in a previously HIV-negative or HSV-2 negative participant. Women who were pregnant or had Pap smears with malignant or severe inflammation non-responsive to treatment were excluded and referred to the district hospital in Nellore. Women who tested positive for HIV were treated at the YRG CARE clinic.

Once enrolled, participants were instructed to insert one applicator of hydroxyethylcellulose (HEC) placebo gel, once per day, every day, and to return to the clinic for four monthly follow-up visits.

### 2.3 Behavioral Data Collection

In addition to the WTP survey, a screening behavioural interview was administered to collect data on demographics, HIV-risk behaviors, sexual and reproductive health, including self-reported gynecological symptoms, reproductive tract surgeries and family planning methods used.

The study was approved by the Institutional Review Boards of the Population Council and YRG CARE, and the Health Ministry Screening Committee through the Indian Council for Medical Research for both the behavioral data collection and placebo microbicide trial. Written informed consent was obtained from all participants before screening and enrollment.

## 3. Data Collection and Management

The WTP survey and behavioral interview responses used a combination of face-to-face (FTF) interviews captured on paper case report forms (CRFs) for socio-demographic variables, and audio computer assisted self-interviews (ACASI) for data on sex work, HIV risk behaviours, and gel and condom use. All clinical and laboratory data were entered onto CRFs. CRFs were sent via DataFax (Data Management Software for Clinical Trials, Clinical DataFax Systems, Inc., Ontario, Canada V3.7) and ACASI data were securely uploaded to the Population Council in New York.

## 4. Statistical Analysis

All analyses were conducted using SAS Version 9.2 (Cary, USA) for Windows. Unpaired Student’s *t* test and the Mann-Whitney *U* test compared continuous variables with normal or non-normal distributions respectively, and a chi-square test identified associations between categorical variables. Logistic regression analysis was undertaken to determine predictors of two outcomes: viral STIs (HIV and HSV-2) and all STIs (viral and bacterial STIs, including HIV, HSV-2, CT, NG, syphilis and TV). Two separate models were fitted for the two outcomes. Variables that were significant at alpha level 0.05 in univariate regression were advanced into the multivariate models using a forward stepwise selection procedure with an entry at p=0.05 and exit at p=0.10. Self-reported condom use was included in both multivariate models, regardless of association on univariate analysis, based on previous evidence of the link between condom use and STI incidence (Nagelkerke et al., 2002).

## 5. Results

A total of 551 of the 730 FSWs who participated in the WTP survey (75.4%) were screened for the trial. Of those, 96.0% (n=529) completed all screening procedures, 48.4% (n=267) enrolled, 33.6% (n=185) were ineligible (pregnant, HIV-positive, pelvic examination not possible, pap smear result showing malignant changes, genital growth/lesion requiring malignancy evaluation), and 17.9% (n=99) were eligible, but did not enroll. Of those enrolled 79.0% (n=211/267) completed the study; details about recruitment (Tun et al., 2011), willingness to participate (Mensch et al., 2012), and participant disposition are reported elsewhere (Abbott et al., 2012).

The mean age of FSWs screened was 30.9 years (SD: 6.5), 68.6% were currently married or cohabiting, and 62.6% reported no education (Table 1). More than half practiced sex work from their homes and one-third started practicing sex work before 15 years of age. The majority of FSWs reported debt, and about half drank alcohol and had experienced sexual violence or physical abuse in the month before screening.

**Table 1:** Characteristics of female sex workers screened for a placebo microbicide clinical trial in Nellore, Andhra Pradesh (N=551)

	% (n)
**Age (n=538)^1^**	
<25 years	16.7% (90)
25-35 years	42.9% (231)
≥36 years	40.3% (217)
**Marital Status (n=538)**^1^	
Single/never married	0.2% (1)
Married/cohabiting	68.8% (370)
Widowed/divorced/separated	31.0(167)
**Education (n=538)**^1^	
No education/illiterate	62.6% (337)
Classes 1-10	36.6% (197)
Pre-university (high school)	0.6% (3)
University	0.2% (1)
**Religion (n=538)**^1^	
Hindu	71.7% (386)
Muslim	11.7% (63)
Christian	16.5% (89)
**Type of Sex Worker (n=543)**^2^	
Home based	57.5% (312)
Brothel based	27.6% (150)
Public place	14.9% (81)
**House ownership (n=551)**^2^	
Owns house/flat	58.4% (322)
Does not own a house	41.6% (229)
**Socio-economic status (n=550)**^2,3^	
SES tertile 1	37.5% (206)
SES tertile 2	31.5% (173)
SES tertile 3	31.1% (171)
**Debt status (n=550)**^2^	
Does not owe any money	7.8% (43)
Owes 1- 10,000 Rupees	92.2% (507)
**Alcohol consumption (n=489)**^2^	
Ever drinks alcohol	47.4% (232)
Never drinks alcohol	52.6% (257)
**Experienced sexual violence (n=527)^1,4^**	53.7% (283)
**Experienced physical abuse (n=529)^1,4^**	49.0% (259)
**First client at =<15 years of age**	30.1% (162)
**Self-reported ever condom use (n=527)^1,4,5^**	89.6% (472)
**Anal sex with clients (n=523)^1,4^**	56.2% (294)

1 Data missing due to incomplete screening visit (n=13)

2 Data missing due to missing/incomplete WTP survey (n=8)

3 Socio-economic status was derived from reported access to 14 household goods or services, including toilet facilities, electricity, and television. Using principal component analysis, a composite index was generated based on relative value of assets and categorized into SES tertiles

4 Data reported for the month prior to screening

5 Ever used condom in last month (consistently or inconsistently) with paying /non-paying partner

Nearly two-thirds (62.4%) of FSWs reported no gynecological symptoms at screening (Table 2). Abnormal vaginal discharge was the most common self-reported symptom (31.6%). Almost three-fourths of FSWs reported past gynecological surgery, primarily female sterilization (86.8%); over 10% reported hysterectomy. Nearly all participants reported at least one live birth with a mean of 2.1 children (SD 1.1). Female sterilization was the most frequently reported contraceptive method; median age of sterilized participants was 32 years (IQR 27-36). Temporary contraceptive methods consisted mainly of male condoms. Self-reported condom use with paying or non-paying partners was lower among sterilized participants than non-sterilized participants (88% versus 97%; OR: 0.282; 95%CI: 0.08-0.93); 47.2% of screened participants reported always using condoms, 42.3% used them sometimes and 10.4% never used condoms with partners in the last month [data not shown].

**Table 2:** Self-reported gynaecological/obstetric conditions and contraceptive use among female sex workers who completed screening for a placebo microbicide clinical trial in Nellore, Andhra Pradesh (N=529)

**Gynaecological Symptoms^1^**	
No symptoms reported	62.4% (330)
Abnormal Vaginal Discharge	31.6% (167)
Genital Itching	3.4% (18)
Painful Intercourse	2.7% (14)
Uterine Prolapse/Uterine Mass	2.1% (11)

**Gynaecological Surgery^1^**	73.2% (387)
Tubectomy	86.8% (336)
Hysterectomy	10.6% (41)
Caesarean section/Fibroidectomy	9.3% (36)

**Contraceptive Methods**	
Permanent methods^2^	71.5% (378)
Temporary methods^3^	14.7% (78)
No contraceptive method	13.8% (73)
**Participants with at least 1 live birth**	92.3% (488)

1 More than one choice possible

2 Includes female (n=336) and male sterilization (n=1) and hysterectomies (n=41)

3 Includes oral contraceptive pills (n=1), female condoms (n=2) and male condoms (n=75)

Among FSWs screened for the trial, viral STIs, mainly HSV-2, were more prevalent than bacterial STIs (61.4% [n=325] vs. 18.1% [n=96]) (data not shown). While HSV-2 was most common (60.7%), the antibody test did not distinguish between acute and chronic infection (Table 3). Of the FSWs who were positive for HSV-2 antibodies, only 25% (82/321) had epithelial findings, which were primarily non-specific erythema (79.1%); vesicles, bullae, ulcers or pustules signifying active lesions were observed among 10.9% of women. HIV infection was detected in 5.3% of FSWs, of whom the majority (24/28) were co-infected with HSV-2. TV was the most commonly detected STI (15.5%). RTIs were more frequent than bacterial STIs; 27.8% had symptomatic BV and 18.9% had symptomatic candidiasis. Nearly two-thirds of women (n=344) had both a viral and bacterial STI. During the trial, new STI and RTIs included six cases of TV, four cases of syphilis, three cases of BV, one case each of HSV-2 and CT but no new HIV infections (data not shown).

**Table 3:** Sexually transmitted infections, reproductive tract infections and pelvic examination findings among female sex workers who completed screening for a placebo microbicide clinical trial in Nellore, Andhra Pradesh (N=529)

**Sexually Transmitted Infections**	
Herpes Simplex Virus 2 (HSV-2)^1,2^	60.7% (321)
Trichomonas vaginalis^3^	15.5% (77)
Human Immunodeficiency Virus (HIV)^2^	5.3% (28)
Treponema pallidum (syphilis)^2^	2.8% (15)
Chlamydia trachomatis^4^	2.2% (10)
Neisseria gonorrhoeae^4^	0.7% (3)

**Reproductive Tract Infections**	
Bacterial Vaginosis (BV)^5^	27.8% (138)
Candidiasis^5^	18.9% (94)

**Genital Abnormalities (n=495)**^6^	
Vaginal Discharge	50.7% (251)
Cervical Discharge^7^	5.3% (24)
Adnexal Mass^8^	0.6% (3)
Uterine /Vaginal Wall Prolapse^8^	2.6% (13)
Cervical Mass/Nodule/Vesicles^8^	2.2% (11)
Genital Warts^8^	1.4% (7)
Adnexal Mass^9^	0.6% (3)

**Epithelial Findings (n=134)**	
Erythema	79.1% (106)
Vesicles/Bullae	6.0% (8)
Warts/Condyloma	5.2% (7)
Papule/Plaque/Nodule/Polyp	4.5% (6)
Other EFs	5.2% (7)

**Papanicolaou (Pap) Test Result (n=441)**^10^	
Inflammatory	46.0% (203)
Malignant	1.1% (5)
Normal	45.8% (202)
Insufficient	7.0% (31)

1 Indeterminate HSV results not included (n=9)

2 Based on number of tests performed (n=529)

3 Based on number of tests performed (n=496)

4 Based on number of tests performed (n=446)

5 Based on number of tests performed (n=266); test not clinically indicated (n=232)

6 Missing data due to incomplete screening visit, pregnancy, menses and participant refusal (n=56)

7 Women with no cervix excluded (n=41)

8 n=496

9 n=494

10 Pap smear test not performed (n=110): participant refused (n=14), pregnancy (n=4), hysterectomy (n=41), incomplete screening visit (n=22), menses (n=18), prolapse (n=4), unable to see cervix due to obesity/atrophic vagina (n=5), no reason indicated (n=2)

Vaginal discharge was detected in 50.7% of screened women and cervical discharge in 5.3% (Table 3). Vaginal/cervical discharge was observed in 75% (117/258) of participants who self-reported vaginal discharge and 41.5% (141/258) of those who did not report vaginal discharge [data not shown]. Vaginal discharge was detected in nearly all women with BV (96.4%) and approximately two-thirds of women with bacterial STIs (66.2%). Among women with HSV-2, 66% self-reported having vaginal discharge, yet only 46.4% were found to have vaginal/cervical discharge on clinical exam.

While 11 (2.1%) participants reported uterine/vaginal wall prolapse (see Table 2), prolapse was observed in 13 (2.6%) of participants. The mean age for these participants was 35.3 years (SD 4.9) and the mean parity 4 (SD 2.7). Almost half of FSWs (46%) had Pap smear results indicating inflammatory changes. A total of 134 different epithelial findings were documented among 121(22.8%) participants, with non-specific erythema being the most common.

We explored predictors for viral STIs among the FSWs screened for enrollment into the trial. Variables associated with viral STIs on univariate analysis are presented in Table 4. In multivariate analysis, having a bacterial STI (AOR: 3.62, 95%CI: 1.94-6.77), not owing money (AOR: 2.72, 95%CI: 1.10-6.72), drinking alcohol (AOR: 1.65, 95%CI: 1.07-2.53) and reporting anal sex in the past month (AOR: 1.59, 95%CI: 1.03-2.45) were independent predictors for viral STIs. Being married (AOR: 0.57, 95%CI: 0.35-0.91), ever using a condom (AOR: 0.47, 95%CI: 0.22-1.00), being 25-35 years of age (AOR: 0.39, 95%CI: 0.25-0.63) or being less than 25 years of age (AOR: 0.19, 95%CI: 0.10-0.36) had a protective effect.

**Table 4:** Predictors of viral STIs and all STIs among 551 female sex workers screened for participation in a placebo microbicide trial in Nellore, India

	Model 1: HIV / HSV				Model 2: All STIs			

	OR	P	AOR	P	OR	P	AOR	P
No bacterial STIs (n=433)	Ref	<0.0001	Ref					
Bacterial STI (n=96)	3.02(1.76-5.17)		3.62 (1.94-6.77)	<0.0001				
Aged <25 yrs (n= 90)	0.24(0.14-0.41)	<0.001	0.19 (0.10-0.36)	<0.0001	0.22 (0.13-0.38)	0.0002	0.19(0.10-0.34)	<0.0001
Aged 25-35 yrs (n= 231)	0.45(0.29-0.67)	<0.001	0.392 (0.25-0.63)	<0.0001	0.45 (0.29-0.68)	<0.0001	0.41(0.25-0.66)	0.8064
Age >35 yrs (n= 217)	Ref		Ref		Ref		Ref	
Any education (n= 201)	Ref				Ref			
No education (n=337)	1.76(1.22-2.52)	0.0022	--	--	1.74 (1.21-2.51)	0.0031	--	--
Christian/Muslim (n=152)	Ref				Ref		Ref	
Hindu (n=386)	1.66(1.13-2.44)	0.009	--	--	1.93(1.31-2.85)	0.001	2.14(1.37-3.35)	0.0009
Not married (n=169)	Ref		Ref		Ref		Ref	
Currently married (n=369)	0.46(0.31-0.69)	0.0002	0.57 (0.35-0.91)	0.0183	0.48 (0.32-0.73)	0.0006	0.52(0.32-0.85)	0.0080
Temporary contraceptive method (n=78)	Ref				Ref			
No method (n=73)	1.99(1.04-3.81)	0.0387	--	--	1.89 (0.99-3.62)	0.0556	--	--
Surgical method (n=378)	2.10(1.28-3.44)	0.0031			2.47 (1.51 -4.06)	0.0003		
Owes money (n=507)	Ref		Ref		Ref			
Does not owe money (n=43)	2.29(1.07-4.92)	0.0333	2.72 (1.10-6.72)	0.0302	2.71 (1.17-6.25)	0.0196	--	--
Never uses condoms (n=55)	Ref		Ref		Ref		Ref	
Ever uses condoms (n=472)	1.00(0.56-1.78)	0.9946	0.470 (0.22-1.00)	0.0489	0.99 (0.55-1.79)	0.9844	0.56(0.26-1.17)	0.1202
No forced sex, past month (n=244)	Ref				Ref			
Forced sex, past month (n=283)	1.51(1.06-2.15)	0.0218	--	--	1.43 (1.00-2.04)	0.0532	--	--
No anal sex, past month (n=229)	Ref		Ref		Ref			
Anal sex, past month (n=294)	1.52(1.07-2.18)	0.0203	1.59 (1.03-2.45)	0.0368	1.41 (0.98-2.03)	0.0616	--	--
Home-based FSW (n=312)	Ref				Ref			
Street/Brothel-based FSW (n=231)	0.65(0.45-0.93)	0.0199	--	--	0.72 (0.50-1.03)	0.0732	--	--
First sex >15 yrs old (n=377)	Ref				Ref			
First sex <15 years old (n=162)	1.61(1.08-2.41)	0.0189	--	--	1.49 (0.99-2.24)	0.0553	--	--
Never drinks alcohol (n=257)	Ref		Ref		Ref		Ref	
Ever drinks alcohol (n=232)	1.89(1.30-2.77)	0.0010	1.65 (1.07-2.53)	0.0221	2.05 (1.39 -3.02)	0.0003	1.99(1.30-3.05)	0.0016

**Family planning:** Temporary methods include intrauterine devices, oral contraceptive pills, barrier methods, hormonal implants and rhythm method; permanent methods consist of male/female sterilization and hysterectomy. **Condom use:** categorized as a binary variable, ever used condoms (consistently or inconsistently) with paying or non-paying partners in the last month or never used condoms with paying or non-paying partners in the last month. **Alcohol consumption:** categorized as a binary variable - ever consumed alcohol and never drinks alcohol

We also explored predictors for the combination of bacterial and viral STIs. On univariate analysis, all variables associated with viral STIs were also associated with both viral and bacterial STIs. In multivariate analysis, belonging to the Hindu faith (AOR: 2.14, 95%CI: 1.37-3.35) and drinking alcohol (AOR: 1.99, 95%CI: 1.30-3.05) were independent predictors; being less than 25 years of age (AOR: 0.19, 95%CI: 0.10-0.34) or 25-35 years of age (AOR: 0.41, 95%CI: 0.25-0.66) or being married (AOR: 0.52, 95%CI: 0.32-0.85) continued to have a protective effect.

## 6. Discussion

This study provides information on the prevalence and incidence of STIs and RTIs and other RH morbidities in a FSW cohort screened for a four-month placebo vaginal gel trial in Nellore, India. The prevalence of HSV-2 detected among FSWs screened for this study was similar to results reported in the Integrated Behavioral and Biological Assessment (IBBA-round 2) survey conducted among FSWs in southern India in 2009-2010, in which HSV-2 prevalence was 52.5% and 61.0% in Chitoor and Prakasham, two districts neighbouring Nellore (IBBA, 2011). In contrast, HIV prevalence was only 5.3% in this study compared to the 10.5% and 13.4% prevalence rates reported for Chitoor and Prakasham (IBBA, 2011). Ulcerative STIs, including HSV-2, are associated with a higher risk for contracting HIV infection (Boily, Baggaley, & Masse, 2009); active herpetic lesions were seen in 10.9% of FSWs with HSV-2 antibodies in our study. More research is necessary to understand the dynamics of HIV transmission in this group with high HSV-2 prevalence, yet low HIV prevalence. It is possible that condoms are more protective against HIV infection, as HSV-2 shedding could take place from genital areas not protected by condoms. Prevalence of syphilis was also lower than what has been reported for other districts in AP (2.9-17.9%) (IBBA, 2011).

While there were a small number of new bacterial STIs observed at the end of a relatively short period of follow-up, there were no new HIV infections. Less than half of the FSWs reported consistent condom-use with paying and non-paying partners in the last month. Low HIV prevalence within the client and primary partner population could possibly be contributing factors (Subramanian et al., 2008).

We also documented high levels of RH morbidity, which has significant implications for future microbicide trials as well as RH programs. A third of the screened participants were found to be ineligible based on gynecological conditions. Pelvic examinations detected vaginal/cervical discharge in 50.7% of screened FSWs; this was higher than the self-reported vaginal discharge. A number of abnormal Pap smears and epithelial findings were also observed highlighting the need for physical examination and Pap smear testing prior to enrolling participants. More importantly, 13 participants had various degrees of vaginal/uterine prolapse and 41 participants had undergone hysterectomy. While these numbers highlight the need for better RH and obstetric care in this population, it also has important implications for future microbicide studies that propose to use vaginal rings or pessary formulations for dispensing gel; to be retained in the vagina, vaginal rings require strong, intact vaginal walls around the cervix. The frequency of hysterectomy in a cohort of reproductive aged women needs further investigation. With the programmatic emphasis on HIV prevention, other RH needs of this population are not being addressed.

AP has the highest rate of female sterilization in the country (International, 2007) and similarly high rates of female sterilization were observed among the FSWs in our study. Sterilized participants were less likely to use condoms with paying and non-paying partners (Wayal et al., 2011). Researchers and HIV prevention advocates should be cognizant of the intersection between sterilization and condom use.

Two-thirds of the FSWs in our study were married, and based on modeling, being married offered significant protection against both viral and bacterial STIs; married FSWs had fewer partners (data not shown). A study exploring the correlates of HIV prevalence among FSWs attending STI clinics in Pune and the IBBA survey also report a higher probability of HIV infection among widowed and single FSWs, similar to our results (Brahme et al., 2006; Ramesh et al., 2008). The IBBA survey found a higher risk for HIV infection among younger FSWs (<25 years) (Ramesh et al., 2008). However, younger FSWs in our study were at lower risk for STIs; perhaps this is because they have engaged in sex work for fewer years and thus have been exposed for a shorter period of time. Vulnerability to STI infection was strongly associated with alcohol consumption and self-reported anal sex; while the association between STI infection and alcohol consumption has been reported in other studies, we did not find any recent studies reporting the connection between vaginal STIs and anal sex (Saggurti et al., 2012). An understanding of these factors is essential to guide inclusion criteria when planning a future microbicide effectiveness study.

Further, although HIV prevalence was low among FSWs screened for this study, they were economically impoverished, reported high levels of sexual and physical violence and had high reproductive health morbidity. The Targeted Intervention program under NACO (2011) focuses mainly on HIV prevention and STI management. HIV prevention programs need to expand coverage to include provision of comprehensive RH services for FSWs.

The study was limited in that the four-month follow-up period in this study was much shorter than that of Phase 2/3 trials. Longer follow-up would have permitted better assessment of incident infections, especially HIV. While indicative of risk, a few new infections occurred during the trial, those detected were predominantly bacterial STIs. In addition, the study sample may not be representative of the wider FSW population in Nellore as the participants in this study self-selected and agreed to be screened for and enroll in a clinical trial (Mensch et al., 2012).

## 7. Conclusion

Thus, although there is high HSV-2 prevalence, an infection that newer generation microbicides aim to target, the low HIV prevalence and incidence and high RH morbidity make this population unsuitable for a Phase2/3 microbicide efficacy trial. Comprehensive RH services for FSWs should be included within targeted HIV prevention programs in India.
